# *Alternaria solani* effectors AsCEP19 and AsCEP20 reveal novel functions in pathogenicity and conidiogenesis

**DOI:** 10.1128/spectrum.04214-23

**Published:** 2024-06-24

**Authors:** Siyu Xiao, Jinhui Wang, Zihan Bai, Yang Pan, Qian Li, Dongmei Zhao, Dai Zhang, Zhihui Yang, Jiehua Zhu

**Affiliations:** 1 College of Plant Protection, Hebei Agricultural University, Baoding, Hebei, China; 2 Technological Innovation Center for Biological Control of Crop Diseases and Insect Pests of Hebei Province, Baoding, Hebei, China; Universita degli Studi del Molise, Campobasso, Italy

**Keywords:** *Alternaria solani*, AsCEP19 and AsCEP20, conidiogenesis, pathogenicity, subcellular localization, weighted gene co-expression network analysis

## Abstract

Previous work identified a pair of specific effectors AsCEP19 and AsCEP20 in *Alternaria solani* as contributors to the virulence of *A. solani*. Here, we constructed *AsCEP19* and *AsCEP20* deletion mutants in *A. solani* strain HWC168 to further reveal the effects of these genes on the biology and pathogenicity of *A. solani*. Deletion of *AsCEP19* and *AsCEP20* did not affect vegetative growth but did affect conidial maturation, with an increase in the percentage of abnormal conidia produced. Furthermore, we determined the expression patterns of genes involved in the conidiogenesis pathway and found that the regulatory gene *abaA* was significantly upregulated and *chsA*, a positive regulator for conidiation, was significantly downregulated in the mutant strains compared to the wild-type strain. These results suggest that AsCEP19 and AsCEP20 indirectly affect the conidial development and maturation of *A. solani*. Pathogenicity assays revealed significantly impaired virulence of Δ*AsCEP19*, Δ*AsCEP20,* and Δ*AsCEP19 + AsCEP20* mutants on potato and tomato plants. Moreover, we performed localization assays with green fluorescent protein-tagged proteins in chili pepper leaves. We found that AsCEP19 can specifically localize to the chloroplasts of chili pepper epidermal cells, while AsCEP20 can localize to both chloroplasts and the plasma membrane. Weighted gene co-expression network analysis revealed enrichment of genes of this module in the photosynthesis pathway, with many hub genes associated with chloroplast structure and photosynthesis. These results suggest that chloroplasts are the targets for AsCEP19 and AsCEP20.

**IMPORTANCE:**

*Alternaria solani* is an important necrotrophic pathogen causing potato early blight. Previous studies have provide preliminary evidence that specific effectors AsCEP19 and AsCEP20 contribute to virulence, but their respective functions, localization, and pathogenic mechanisms during the infection process of *A. solani* remain unclear. Here, we have systematically studied the specific effectors AsCEP19 and AsCEP20 for the first time, which are essential for conidial maturation. The deletion of AsCEP19 and AsCEP20 can significantly impair fungal pathogenicity. Additionally, we preliminarily revealed that AsCEP19 and AsCEP20 target the chloroplasts of host cells. Our findings further enhance our understanding of the molecular mechanisms underlying the virulence of necrotrophic pathogens.

## INTRODUCTION

*Alternaria solani* is a necrotrophic pathogen that causes global diseases, including potato and tomato early blight (1–3). Infection with *A. solani* can cause black lesions with a yellow halo and a concentric ring symptom on the crop leaves, affecting the overall plant growth (4, 5). Therefore, there is important incentive to understand the pathogenic mechanism of *A. solani* to develop effective control strategies.

Unlike biotrophic and hemibiotrophic pathogens, necrotrophic pathogens actively induce plant tissue cell death and directly obtain nutrition from dead cells (6–8). These pathogens secrete non-host-selective toxins, host-selective toxins (9), or plant cell wall degrading enzymes to participate in the pathogenic process of pathogens. Among them, the relationship between the genus *Alternaria* toxins and pathogenicity has been the most clearly studied. Tenuazonic acid, alternariol, alternariol monomethyl ether, altenuene, and the perylene derivative altertoxins (ATX-I) are the main *Alternaria* toxins (10, 11). Many non-host-selective toxins and host-selective toxins secreted act on chloroplasts, mitochondria, and plasma membranes, and can suppress host defense responses to induce host cell susceptibility (12, 13). Different pathogenic *Alternaria* fungi can use horizontal transfer to obtain additional small chromosomes that carry synthetic gene clusters of host-specific toxins (14). One host-specific toxin, alternaric acid, has been isolated from *A. solani* and has antifungal effects (15). In the presence of Ca^2+^and Mg^2+^, alternaric acid can stimulate the phosphorylation of His-tagged calcium-dependent protein kinase 2 from potato cultivar Rishiri to suppress hypersensitive cell death (16).

With the recent rapid development of multiomics, more and more effector proteins secreted by necrotrophic pathogens have been identified that contribute to the virulence of pathogens. *Sclerotinia sclerotiorum* secretes several characterized proteinaceous effectors, including SsINE5, which induces host cell death via an NLR protein (17), and SsERP1, which interacts with host polygalacturonase-inhibiting proteins to enhance pathogenic virulence (18). A cytoplasmic effector Crh1 secreted by *Botrytis cinerea* can trigger plant cell death and defense response (19). However, few effectors have been cloned from the necrotrophic pathogenic fungi because the effectors secreted by the necrotrophic pathogens do not exist as gene families or clusters and are often lineage-specific.

In a previous study, 238 effector candidates were predicted in the *A. solani* genome (20), including a non-specific effector AsCEP112 (21) and AsCEP50 (22) and a specific pair of effectors AsCEP19 and AsCEP20 (20). Two lineage-specific effector genes, *AsCEP19* and *AsCEP20*, were found to form a ‘head-to-head’ gene pair located near a base AT-rich region on chromosome 3 (20). This pair of effector genes was likely acquired by the common ancestor of *A. solani* and *Alternaria tomatophila* via horizontal gene transfer (20). *AsCEP19* and *AsCEP20* are tightly co-expressed in a host-specific manner that they are upregulated at advanced stages of *A. solani* infection only in solanaceous hosts, and contribute to the virulence of *A. solani* (20). Although only a few effectors have been identified in *A. solani*, a few specific effectors play an important role in the virulence of *A. solani*. Previous studies have provided preliminary evidence that specific effectors contribute to virulence, but their respective functions, localization, and pathogenic mechanisms during the infection process of *A. solani* remain unclear.

In this study, we constructed mutation strains deleted for effector genes *AsCEP19* and *AsCEP20,* respectively, to further investigate the effects of these effectors on the biology and pathogenicity of *A. solani*. We found that AsCEP19 and AsCEP20 affect conidial maturation and are required for full virulence. Subcellular localization analysis indicated that chloroplasts in the epidermal cells of solanaceous host are potential targets for these two effectors. Furthermore, AsCEP20 mainly affects the photosynthesis pathway. Taken together, these results further our understanding of the molecular mechanisms underlying the virulence of necrotrophic pathogens.

## RESULTS

### Generation of *AsCEP19* and *AsCEP20* deletion mutants and complementation strains

**Fig 1:**
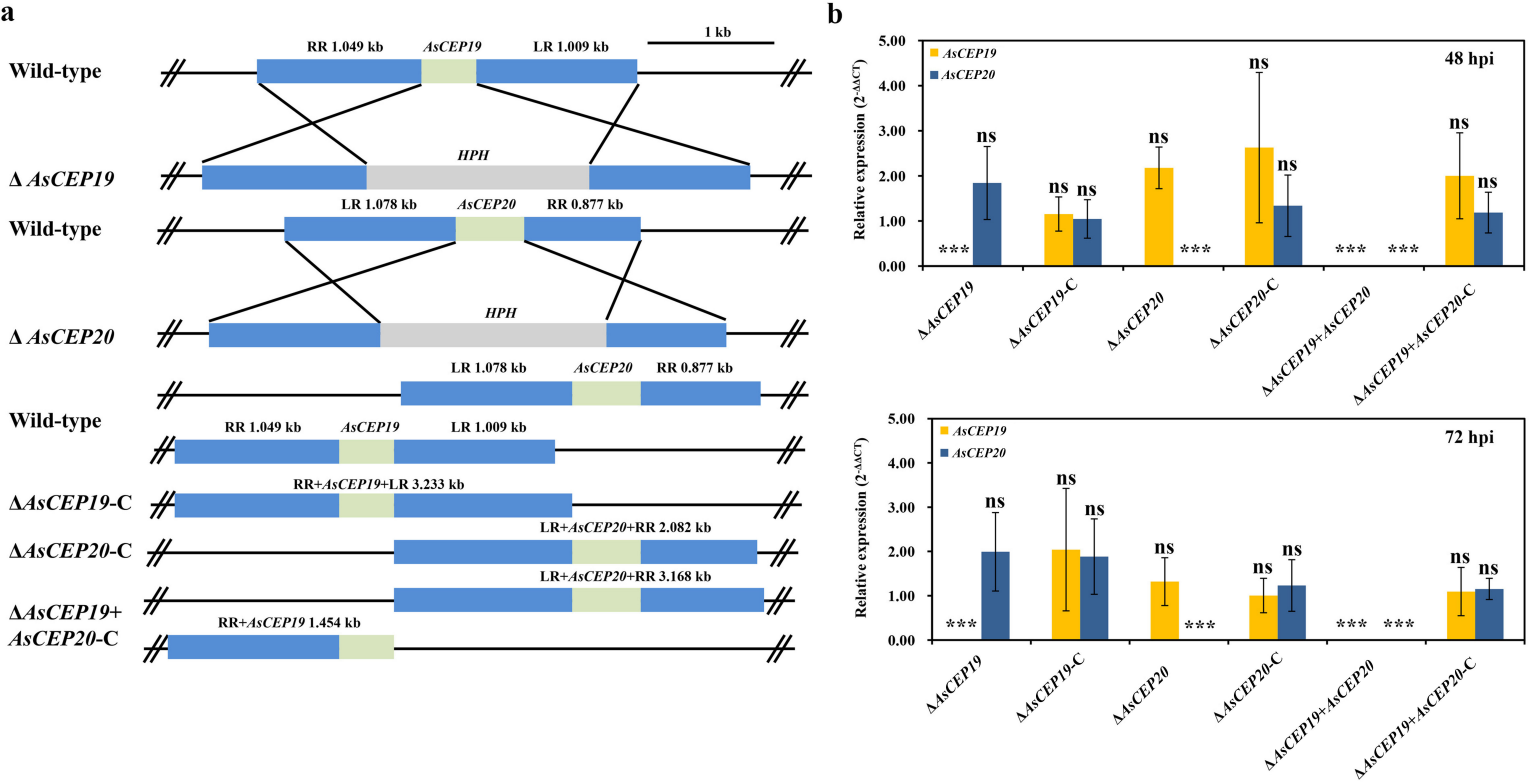
Targeted deletion of *AsCEP19* and *AsCEP20* in *A. solani*. (a) Strategy to generate *AsCEP19* and *AsCEP20* deletion mutants and complementation strains. (b) The expression levels of *AsCEP19* and *AsCEP20* in different mutants at different time points after inoculation were compared with those of *A. solani* WT strains. The β-actin gene (*ACTB*) of *A. solani* was used as an internal reference gene. Error bars represent means ± SD from six biological replicates. Two-tailed Student’s *t*-tests, *P < 0.05, **P < 0.01, and ***P < 0.001, ns means not significant.

To explore the potential effects of the *AsCEP19* and *AsCEP20* genes in *A.solani*, we generated Δ*AsCEP19*, Δ*AsCEP20*, and Δ*AsCEP19 + AsCEP20* mutants using a targeted gene replacement strategy. Hygromycin phosphotransferase gene (*HPH*) was transformed into the *A. solani* strain HWC168 via homologous recombination to replace the *AsCEP19* gene or the *AsCEP20* gene (Fig. 1a). The putative *AsCEP19* and *AsCEP20* deletion mutants and complementation strains were screened by PCR amplification (Fig. S1) and sequencing (Table S1). The expression levels of *AsCEP19* and *AsCEP20* were significantly upregulated during the infection stage compared to the mycelium stage (20). We assayed *AsCEP19* and *AsCEP20* expression in the different strains at 48 and 72 h post inoculation (hpi) by reverse transcription-quantitative PCR (RT-qPCR). No expression of *AsCEP19* was detected in Δ*AsCEP19* and Δ*AsCEP19 + AsCEP20* mutants, with average Ct values of the two genes being 35.87 and 33.76, respectively. *AsCEP20* showed no expression (Ct >37) in Δ*AsCEP20* and Δ*AsCEP19 + AsCEP20* mutants, with average Ct values of 37.75 and 34.72, respectively. The expression pattern of effector genes at 72 hpi was the same at 48 hpi. There was no significant difference in the expression of *AsCEP19* and *AsCEP20* in complementation strains compared to the wild-type (WT) strain at 48 hpi and 72 hpi (Fig. 1b).

### AsCEP19 and AsCEP20 are not involved in *A. solani* vegetative growth

**Fig 2:**
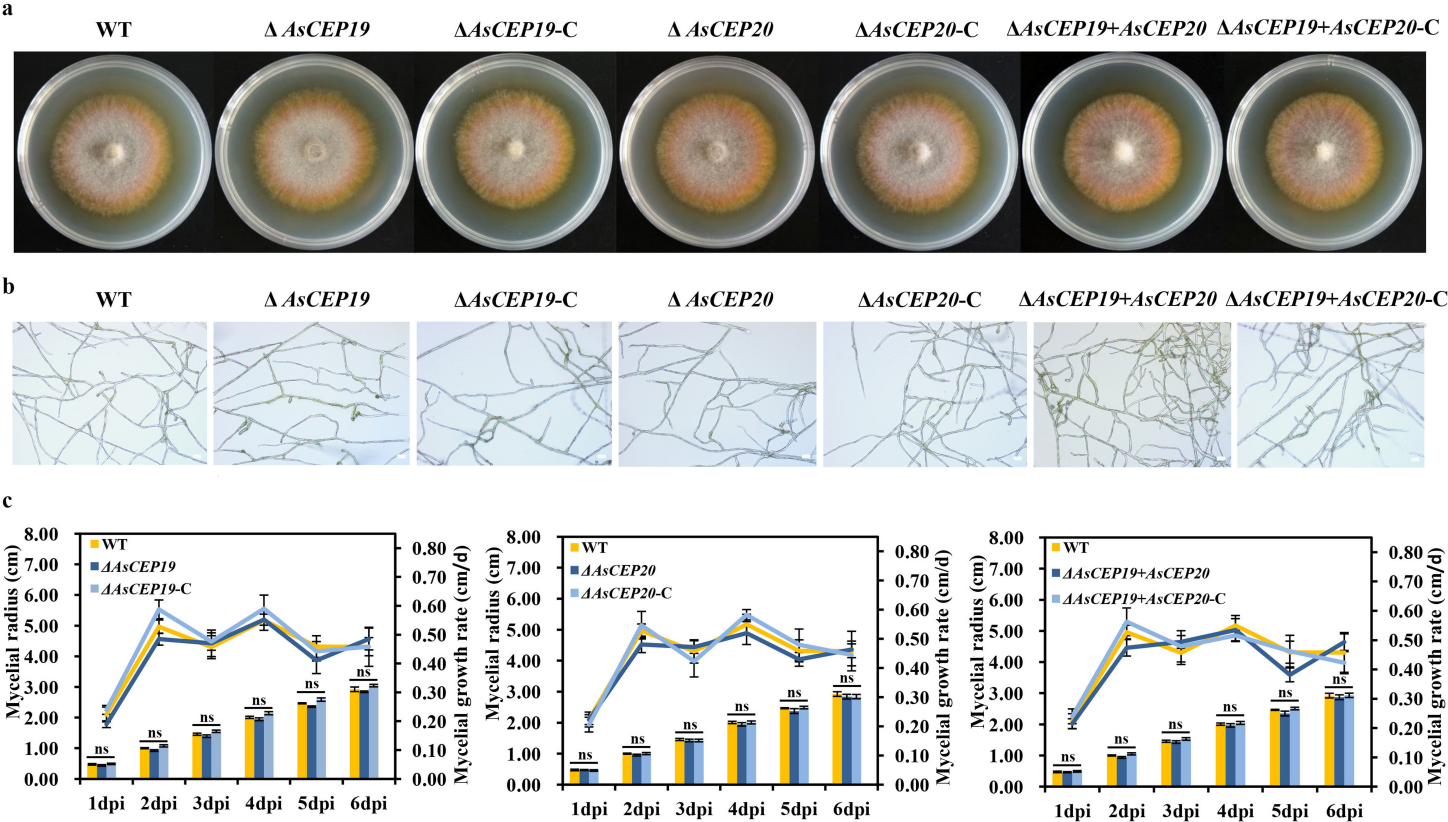
Growth phenotype analysis of *AsCEP19* and *AsCEP20* deletion mutants, complementation strains, and WT. (a) Mycelial morphology of the wild-type, Δ*AsCEP19*, Δ*AsCEP19*-C, Δ*AsCEP20*, Δ*AsCEP20*-C, Δ*AsCEP19 + AsCEP20*, and Δ*AsCEP19 + AsCEP20*-C strains grown on PDA plates at 25°C for 6 days. (b) Microscopic observation of hyphae from the wild-type, Δ*AsCEP19*, Δ*AsCEP19*-C, Δ*AsCEP20*, Δ*AsCEP20*-C, Δ*AsCEP19 + AsCEP20*, and Δ*AsCEP19 + AsCEP20*-C strains grown on PDA plates at 25°C for 2 days. Bar, 20 µm. (c) Mycelial growth rates and radius measurements of all indicated strains grown on PDA plates for 6 days. The mean values ± SD were calculated from three independent experiments, with six repeats for each strain in all independent experiments. Two-tailed Student’s *t*-tests, ns means not significant.

We examined the growth of Δ*AsCEP19*, Δ*AsCEP20,* and Δ*AsCEP19 + AsCEP20* mutants and complementation strains cultured on potato dextrose agar (PDA) plates for 6 days. The mycelia of Δ*AsCEP19*, Δ*AsCEP20,* and Δ*AsCEP19 + AsCEP20* mutants did not differ morphologically from those of the WT and complementation strains (Fig. 2a). Further microscopic examination showed that the hyphae from the Δ*AsCEP19*, Δ*AsCEP20,* and Δ*AsCEP19 + AsCEP20* mutants did not significantly differ in the number of mycelia branches, color, and strength compared those of the WT and the complementation strains (Fig. 2b). In addition, the mycelia radius and mycelial growth rate of the mutants were similar to those of the WT and the complementation strains (Fig. 2c). Taken together, these results indicated that *AsCEP19* and *AsCEP20* do not affect vegetative growth.

### AsCEP19 and AsCEP20 affect conidial maturation in *A. solani*

**Fig 3:**
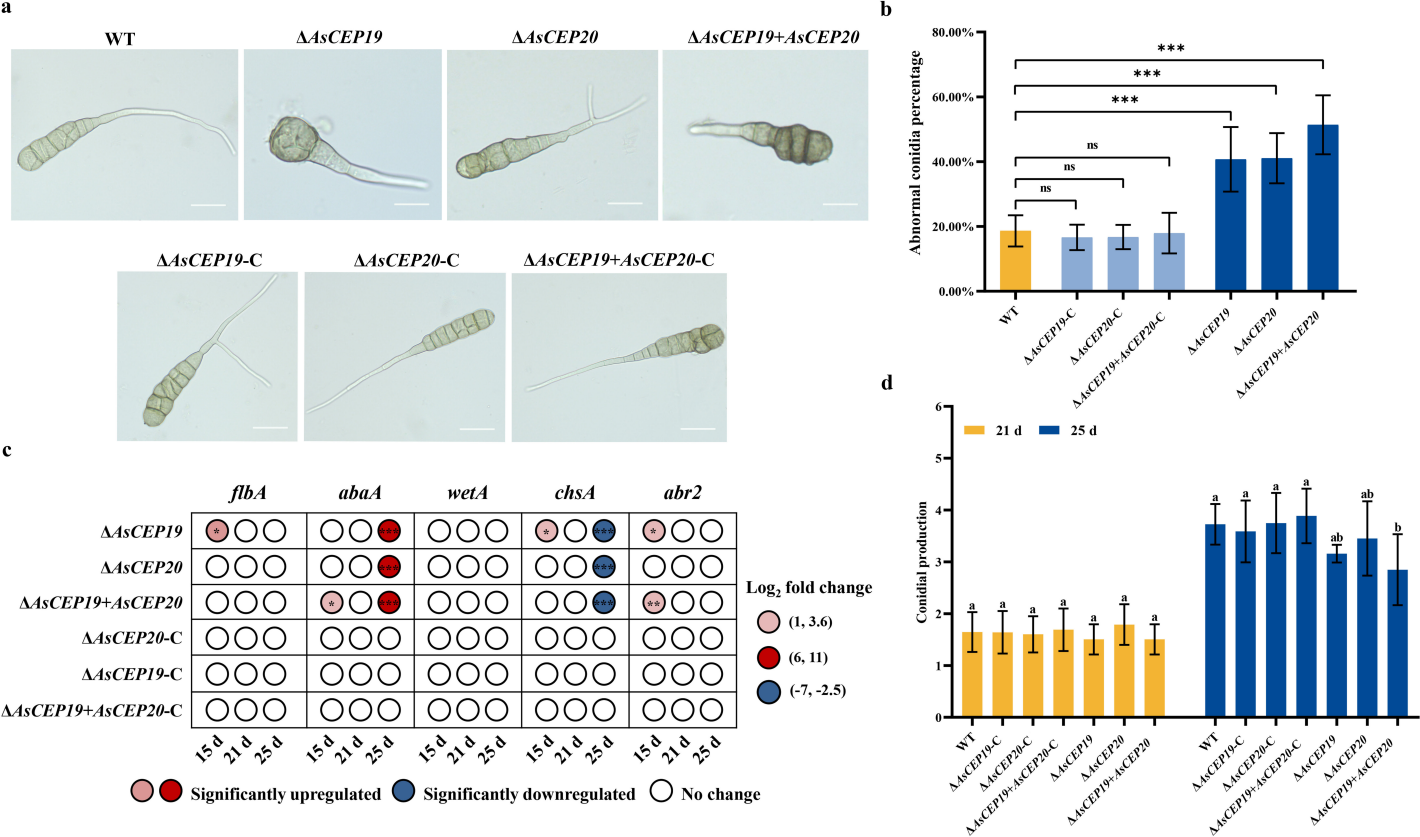
Conidial development and maturation analysis of *AsCEP19* and *AsCEP20* deletion mutants, complementation strains, and the wild-type strain HWC168. (a) Microscopic (400×) observation of conidia morphology and development of the WT, Δ*AsCEP19*, Δ*AsCEP19*-C, Δ*AsCEP20*, Δ*AsCEP20*-C, Δ*AsCEP19 + AsCEP20*, and Δ*AsCEP19 + AsCEP20*-C strains. Bar, 20 µm. (b) The percentages of abnormal conidia in the total number of spores in a field of view. Twenty fields were selected for each strain (based on Wilcoxon test, using the Bonferroni *P*-value adjustment method). (c) The expression levels of conidiogenesis regulatory genes in WT, Δ*AsCEP19*, Δ*AsCEP19*-C, Δ*AsCEP20*, Δ*AsCEP20*-C, Δ*AsCEP19 + AsCEP20*, and Δ*AsCEP19 + AsCEP20*-C strains grown on TA plates at different time points, which were compared with those of *A. solani* WT strains. The *actin* gene of *A. solani* was used as reference gene. Error bars represent means ± SD from six biological replicates. Two-tailed Student’s *t*-tests, *P < 0.05, **P < 0.01, and ***P < 0.001, ns means not significant. The Bonferroni *P*-value adjustment method was used. (d) WT, Δ*AsCEP19*, Δ*AsCEP19*-C, Δ*AsCEP20*, Δ*AsCEP20*-C, Δ*AsCEP19 + AsCEP20*, and Δ*AsCEP19 + AsCEP20*-C strains grown on TA plates at 21 days and 25 days. A 100× microscope was used to count the number of conidia in a field of view. The mean values ± SD were calculated from three independent experiments (*P* < 0.05, based on one-way analysis of variance, Bonferroni *P*-value adjustment method).

Conidia are a common reproduction strategy in *A. solani*. We observed the conidial morphology of *A. solani* to study the effects of *AsCEP19* and *AsCEP20* on the biological function of *A. solani*. Most of the conidia produced by the Δ*AsCEP19*, Δ*AsCEP20,* and Δ*AsCEP19 + AsCEP20* deletion mutants appeared significantly stunted, with or without oblique or longitudinal septa. These conidia are enlarged at the tip, irregular in shape, and have a shorter and thicker filamentous apical beak compared to those of WT and complementation strains (Fig. 3a). There was a 22.09% increase in abnormal conidia percentage for the gene deletion mutantΔ*AsCEP19*, a 22.42% increase for Δ*AsCEP20*, and a 32.73% increase for Δ*AsCEP19 + AsCEP20* than WT (Fig. 3b). Furthermore, we measured the expression of regulatory genes in the conidiogenesis pathway. AsCEP19 and AsCEP20 significantly affected the expression of the *abaA* and *chsA* genes (Fig. 3c). The *abaA* gene is a member the central regulatory pathway that controls sporulation in *Aspergillus nidulans*, and is associated with the formation of supernumerary tiers of cells with abacus-like structures (23, 24). The *chsA* gene is a positive regulator for conidiation in *Aspergillus niger* (25). The expression levels of *abaA* were significantly upregulated, while those of *chsA* were significantly downregulated in the Δ*AsCEP19*, Δ*AsCEP20,* and Δ*AsCEP19 + AsCEP20* mutants compared to the WT at 25 days grown on tomato juice agar (TA) plates (Fig. 3c). The expression levels of *abaA* were 8.04 × 10^2^-fold (Log_2_ fold change was 9.48), 8.54 × 10^2^-fold (Log_2_ fold change was 9.70), and 6.05 × 10^2^-fold (Log_2_ fold change was 8.42) higher in the deletion mutants than those of WT, respectively. The expression levels of *chsA* were 8.77-fold (Log_2_ fold change was −2.87), 2.03 × 10^2^-fold (Log_2_ fold change was −6.99), and 13.14-fold (Log_2_ fold change was −3.42) lower in the deletion mutants than those of WT, respectively. The expression levels of *flbA*, *abaA*, *chsA*, and *abr2* were slightly upregulated at 15 days. Expression level of *flbA* was 3.30-fold (Log_2_ fold change was 1.67) higher in Δ*AsCEP19* than the WT, expression level of *abaA* was 3.99-fold (Log_2_ fold change was 1.96) higher in Δ*AsCEP19 + AsCEP20* than the WT, expression level of *chsA* was 2.66-fold (Log_2_ fold change was 1.27) higher in Δ*AsCEP19* than the WT, and expression levels of *abr2* were 5.90-fold (Log_2_ fold change was 2.37) and 6.79-fold (Log_2_ fold change was 2.60) higher in Δ*AsCEP19* and Δ*AsCEP19 + AsCEP20* than those of WT, respectively (Fig. 3c; Table S2). In addition, we also found that the deletion mutants were reduced in conidial production at 25 days (Fig. 3d). These results indicated that *AsCEP19* and *AsCEP20* are important for conidia formation in *A. solani*.

### *AsCEP19* and *AsCEP20* are required for the full virulence in *A. solani*

**Fig 4:**
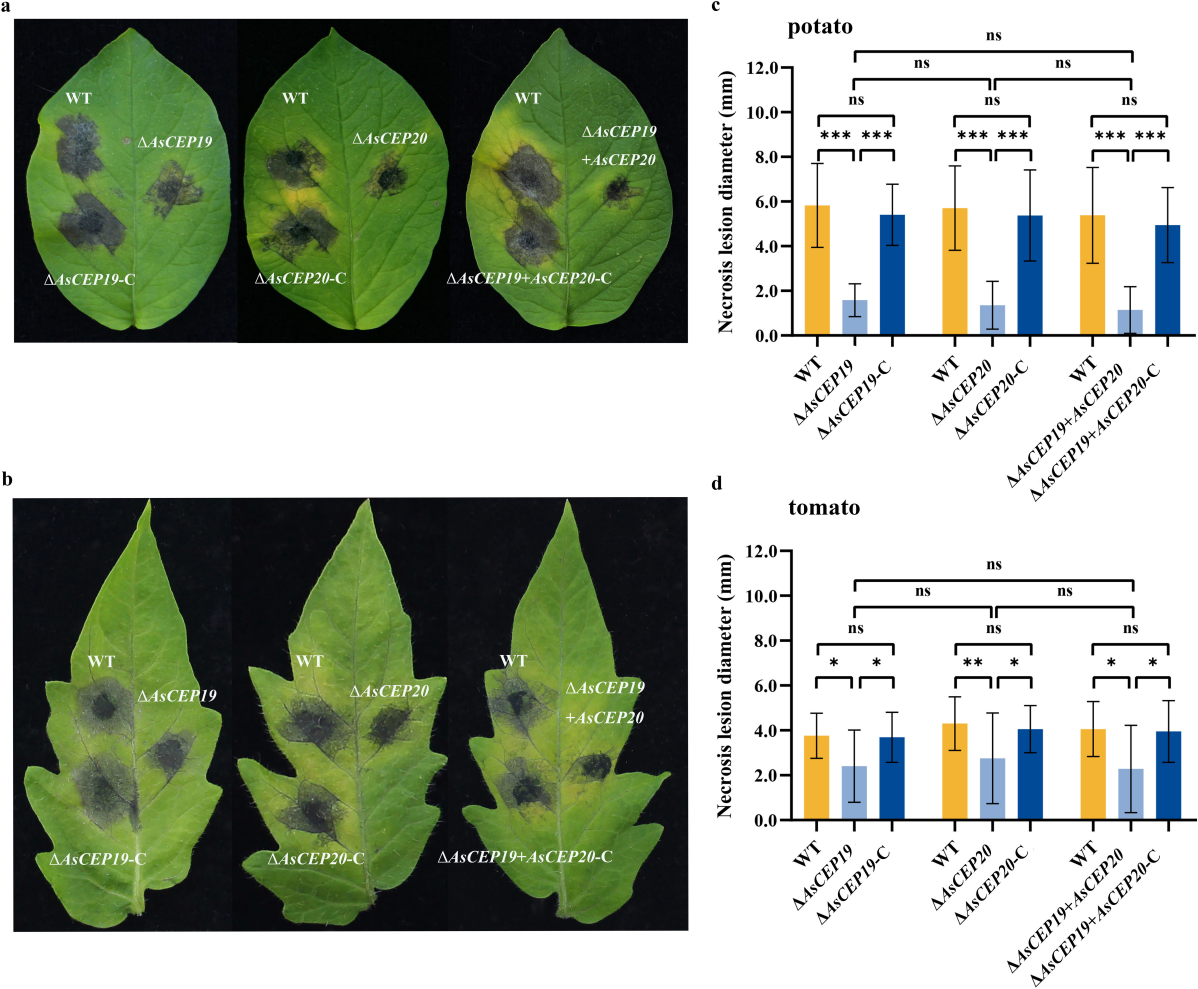
Variation in virulence for *AsCEP19* and *AsCEP20* deletion mutants, complementation strains, and wild-type *A. solani* on potato (**a, c**) and tomato (**b, d**) leaves. Detached leaves were inoculated with conidial suspensions of the wild-type strain (upper left), the complementation strains (lower left), and the deletion mutants (right). The sizes of necrotic lesions were measured at 5 dpi. Error bars indicate the standard deviation of the means, and asterisks indicate statistical significance determined by paired *t*-test (*n* = 30), **P < 0.01 and ***P < 0.001**,** ns means not significant. The Bonferroni *P*-value adjustment method was used.

Deletion of *AsCEP19* and *AsCEP20* from *A. solani* HWC168 reduced virulence on potato leaves (Fig. 4a). The necrotic lesion caused by: the gene deletion mutant Δ*AsCEP19* (1.58 ± 0.74 mm) on potato was much smaller than that of WT (5.82 ± 1.88 mm) and Δ*AsCEP19*-C (5.41 ± 1.37 mm), Δ*AsCEP20* (1.36 ± 1.07 mm) was much smaller than that of WT (5.71 ± 1.89 mm) and Δ*AsCEP20*-C (5.38 ± 2.15 mm), and Δ*AsCEP19 + AsCEP20* (1.14 ± 1.05 mm) was much smaller than that of WT (5.38 ± 2.15 mm) and Δ*AsCEP19 + AsCEP20*-C (4.94 ± 1.68 mm) (Fig. 4c). The same trend was observed for the infection of the mutants on *A. solani* on tomato leaves (Fig. 4b). The necrotic lesion caused by: the gene deletion mutantΔ*AsCEP19* (2.41 ± 1.61 mm) was much smaller than that of WT (3.76 ± 1.00 mm) and Δ*AsCEP19*-C (3.69 ± 1.12 mm), Δ*AsCEP20* (2.75 ± 2.02 mm) was much smaller than that of WT (4.30 ± 1.19 mm) and Δ*AsCEP20*-C (4.05 ± 1.05 mm), and Δ*AsCEP19 + AsCEP20* (2.28 ± 1.95 mm) was much smaller than that of WT (4.06 ± 1.22 mm) and Δ*AsCEP19 + AsCEP20*-C (3.95 ± 1.38 mm). The deletion of *AsCEP19* resulted in a 72.83% and 36.07% reduction in the pathogenicity of *A. solani* in potatoes and tomatoes, respectively. The deletion of *AsCEP20* resulted in a 76.23% and 36.03% reduction in the pathogenicity of *A. solani* in potatoes and tomatoes, respectively. The deletion of *AsCEP19* and *AsCEP20* resulted in a 78.81% and 43.84% reduction in the pathogenicity of *A. solani* in potatoes and tomatoes, respectively. These results show that deletion of *AsCEP19* and *AsCEP20* from *A. solani* HWC168 significantly reduced disease severity. *AsCEP19* and *AsCEP20* contribute significantly to the full virulence of *A. solani* on host potato and tomato plants. Interestingly, there were no significant differences in the necrotic lesion produced by Δ*AsCEP19*, Δ*AsCEP20,* and Δ*AsCEP19 + AsCEP20* (Fig. 4c and d).

### Subcellular localization of AsCEP19 and AsCEP20 in chili pepper

**Fig 5:**
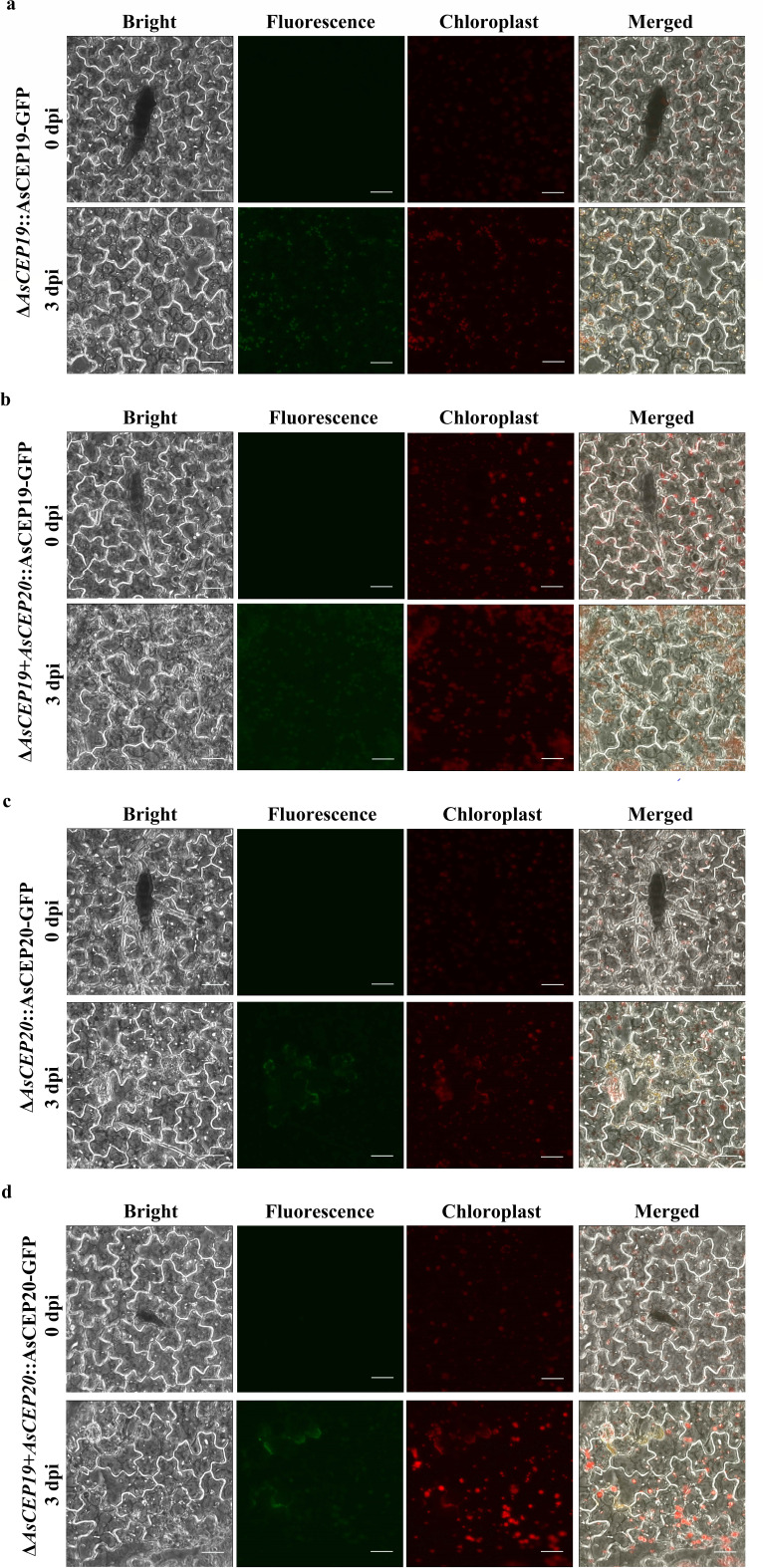
Subcellular localization of AsCEP19 and AsCEP20 in chili pepper leaves. (a) Subcellular localization of AsCEP19-GFP fusion protein in the Δ*AsCEP19* strain during plant infection. (b) Subcellular localization of AsCEP19-GFP fusion protein in the Δ*AsCEP19 + AsCEP20* strain during plant infection. (c) Subcellular localization of AsCEP20-GFP fusion protein in the Δ*AsCEP20* strain during plant infection. (d) Subcellular localization of AsCEP20-GFP fusion protein in the Δ*AsCEP19 + AsCEP20* strain during plant infection. Bar, 30 µm.

Potato leaves are thick, with a narrow distance between leaf veins, making sectional observation difficult. Therefore, we selected chili pepper leaves, a plant closely related to potato, for localization experiments. We monitored the subcellular localization of green fluorescent protein (GFP)-tagged effectors AsCEP19 and AsCEP20 during *A. solani* infection in chili pepper leaves. No GFP fluorescence signals were observed during the initial inoculation of *A. solani*, and the fluorescence signals of AsCEP19 and AsCEP20 were significantly enhanced in *A. solani*-infected plants at 3 dpi (Fig. 5). In addition, with the infection of *A. solani*, the effectors were transferred from the interior of the hyphae to the host cell. In both the presence and absence of AsCEP20, AsCEP19 specifically localized to the chloroplasts of chili pepper epidermal cells (Fig. 5a and b). Similarly, in both the presence and absence of AsCEP19, AsCEP20 localized to chloroplasts, but also observed on the plasma membrane (Fig. 5c and d). Thus, the results show that both AsCEP19 and AsCEP20 are expressed in the chloroplasts.

### Weighted gene co-expression network analysis (WGCNA) of the virulence factor AsCEP20 in potato response to *A. solani*

**Fig 6:**
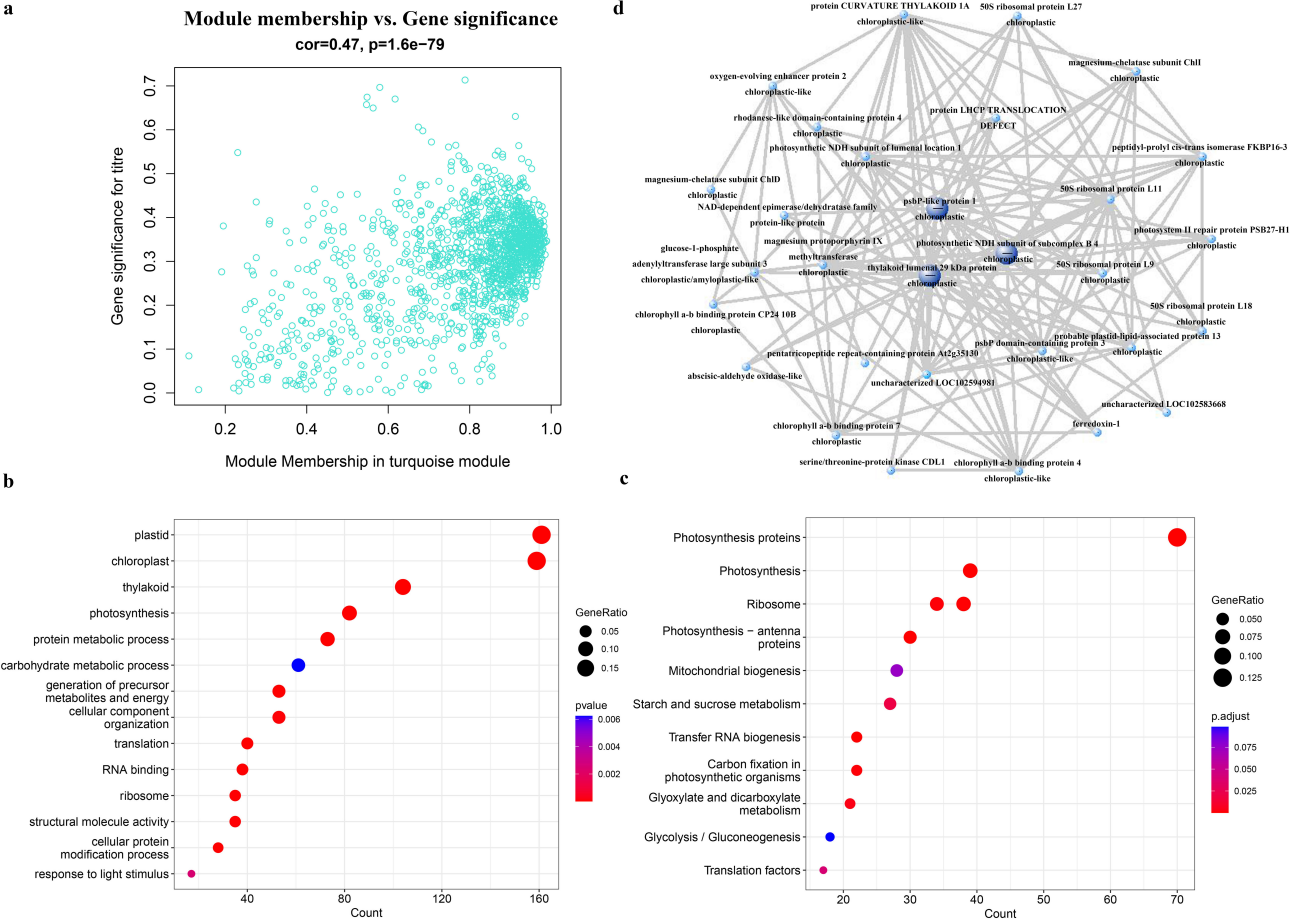
WGCNA of AsCEP20 in *A. solani.* (a) Correlation between turquoise gene module and fungal genotype. (b) GO enrichment analysis of the turquoise gene module. (c) KEGG pathway enrichment analysis of the turquoise gene module. (d) Hub genes co-expression network of the turquoise module.

RNA-Seq data were obtained for different potato varieties inoculated with Δ*AsCEP20* and WT strains. Association analysis of phenotypes such as potato resistance, strain genotype, and potato variety resulted in 20 modules (Fig. S4). Among these 20 different modules, there were 1,431 genes in the turquoise module and this module showed the highest correlation with strain genotype (Fig. 6a; Fig. S4). Analysis of Gene Ontology (GO) categories and Kyoto Encyclopedia of Genes and Genomes (KEGG) pathways showed that these genes are associated with chloroplasts and involved in the photosynthesis pathway (Fig. 6b and c). The top 30 genes with high internal correlation were selected to construct a network, and three hub genes were identified. These three hub genes encode psbP-like protein 1, photosynthetic NAD(P)H dehydrogenase (NDH) subunit of subcomplex B 4, and thylakoid luminal 29 kDa protein (Fig. 6d), all of which are associated with chloroplasts. These results suggested that AsCEP20 function is highly correlated with chloroplasts.

## DISCUSSION

This study analyzed the functions of two effectors AsCEP19 and AsCEP20. Analysis of the deletion mutants of *AsCEP19* and *AsCEP20* genes revealed that neither AsCEP19 nor AsCEP20 affects the vegetative growth of *A. solani*, with no changes in mycelial morphology or mycelial growth rate (Fig. 2). This result is consistent with the results of previous work on the effector AsCEP112 secreted by *A. solani* (21).

The deletion of AsCEP19 and AsCEP20 affected conidia maturation, resulting in an increase in the percentage of abnormal conidia produced by *A. solani* (Fig. 3a and b). The expression patterns of upstream activators (*flbA*), central regulatory genes (*abaA*, *wetA*), and downstream regulators (*abr2*, *chsA*) of the conidiogenesis pathway in *A. solani* were analyzed, and there was consistent expression of *abaA* and *chsA* genes for the Δ*AsCEP19*, Δ*AsCEP20,* and Δ*AsCEP19 + AsCEP20* deletion mutants (Fig. 3c). These three sequentially expressed genes, including *brlA*, *abaA,* and *wetA,* comprise a central regulatory pathway that controls the sporulation in *A. nidulans*, and sequentially regulate conidia formation and maturation in *A. nidulans* (23, 26). The *abaA* gene is a key gene in the central regulatory pathway, acting to induce the production and maintenance of conidia, and activate the *wetA* gene. The absence of *abaA* causes the development of additional layers of cells with structures similar to an abacus (23, 24, 27, 28). The *chsA* gene is a chitin synthase gene in *A. nidulans* and is mainly expressed in the metulae, phialides, and conidia (29–31). Deletion of *chsA* reduces the ability to produce asexual spores (conidia) in *A. nidulans* (32), while acting as a positive regulator of conidiation in *A. niger* (25). *chsA* and *chsE* encode a partially redundant function necessary for conidiophore development in *A. nidulans* (32). A *chsE* null strain did not show a defect in conidiation, but a strain in which both *chsA* and *chsE* were absent affected conidium formation (32). Expression of *chsA* and *chsC* may be regulated by AbaA, BrlA, or MedA at the level of transcription (31). In the necrotrophic pathogen *A. solani*, the *abaA* gene is an intermediate gene hub and its expression level was significantly upregulated in three deletion mutants, but the expression of *chsA* was significantly downregulated at 25 days (Fig. 3c). The *abaA* gene may not only directly affect *chsA* through *wetA*, but may also regulate the expression of *chsA* through effectors. This is an unknown mechanism. Other effectors in other necrotrophic pathogens can affect conidia formation, such as the BcCla4 effector secreted by *Botrytis cinerea* (33).

Deletion of *AsCEP19* and *AsCEP20* reduced the virulence of *A. solani* on the host potato and the solanaceae tomato plant, but the virulence decreased more on potatoes compared to tomatoes (Fig. 4). Thus, we confirmed that these effectors act as virulence factors. There was no significant difference in necrotic lesion size between infections with Δ*AsCEP19*, Δ*AsCEP20,* and Δ*AsCEP19 + AsCEP20* strains (Fig. 4c and d). In addition, previous work found that *AsCEP19* and *AsCEP20* lie adjacent to each other, constituting a bidirectional (head-to-head) gene pair which is located within a small GC-equilibrated region (20). These results confirmed that there is a synergistic effect between AsCEP19 and AsCEP20. Thus, AsCEP19 and AsCEP20 are likely to exert their virulence functions as a whole, rather than act alone.

We observed the subcellular localization of effectors AsCEP19 and AsCEP20 on chili pepper leaves, and found that AsCEP19 specifically localized to the chloroplasts, and AsCEP20 localized to chloroplasts as well as the plasma membrane (Fig. 5). These results differ from what was observed with transient expression of the two effectors in *Nicotiana benthamiana*, where AsCEP20 localized to the chloroplasts, but AsCEP19 showed no obvious positioning signal (20). However, transient expression requires the injection of effectors directly into plant epidermal cells, and this differs from how *A. solani* invade plant cells and secrete effectors in natural conditions. Thus, this study will more accurately reflect the secretion process and localization of effectors in natural conditions.

Chloroplasts play a central role in plant defense and are targeted by pathogen effectors (34). AsCEP20 mainly affects the photosynthesis pathway, with hub genes closely related to the structure or function of chloroplasts (Fig. 6). The psbP-like protein 1 is a homolog of PsbP protein in chloroplasts, and promotes the assembly of photosystem II supercomplexes and optimizes plant adaptability under fluctuating light conditions in *Arabidopsis thaliana* (35). Chloroplast NDH mediates the cyclic electron transport of photosystem I by forming a supercomplex with it to act in photosynthesis (36). Similarly, effector SsITL secreted by *S. sclerotiorum* interacts with the calcium-sensing receptor in chloroplasts to inhibit host resistance (37). An effector MoXYL1A promotes the virulence of *Magnaporthe oryzae* by interfering in the proper function of host chloroplasts (38).

In summary, we have systematically studied the specific effectors, AsCEP19 and AsCEP20, which are secreted by *A. solani*. AsCEP19 and AsCEP20 can significantly impair fungal virulence and affect conidial maturation. AsCEP19 and AsCEP20 do not participate in the vegetative growth of *A. solani*. Moreover, AsCEP19 can specifically localize to the chloroplasts of chili pepper epidermal cells, while AsCEP20 can localize to both chloroplasts and the plasma membrane. Weighted gene co-expression network analysis indicated that AsCEP20 mainly affects the photosynthesis pathway. It is suggested that AsCEP19 and AsCEP20 affect the interaction between pathogens and host plants by targeting chloroplasts.

## MATERIALS AND METHODS

### Construction of Δ*AsCEP19* and Δ*AsCEP20* mutants and complementation strains

Targeted deletion of *AsCEP19* and *AsCEP20* was conducted by the polyethylene glycol (PEG4000)-mediated method (39). To replace the target region with the *HPH* gene, the 5′ flanking region of the *AsCEP19* gene (*AsCEP19LR*) and 3′ flanking region of the *AsCEP19* gene (*AsCEP19RR*) were amplified with primer pairs AsCEP19-LR-F2/R1 and AsCEP19-RR-F1/R1, respectively (Table S1). The 5′ flanking region of the *AsCEP20* gene (*AsCEP20LR*) and 3′ flanking region of the *AsCEP20* gene (*AsCEP20RR*) were amplified with primer pairs AsCEP20-LR-F2/R1 and AsCEP20-RR-F1/R1, respectively (Table S1). The *HPH* gene fragment was amplified from the vector pEASY-HPH with primers HPH-F2/R2 and HPH-F1/R1, respectively (Table S1). All PCR reactions were performed using Super Pfx DNA polymerase (CWBIO, China). The three PCR amplicons were fused in the order “*AsCEP19LR-HPH-AsCEP19RR*” and “*AsCEP20LR-HPH-AsCEP20RR*” and then cloned into a pUC19 vector using the pEASY-Uni Seamless Cloning and Assembly Kit (TransGen, Beijing, China). The fused fragment was subsequently amplified from the recombinant plasmid by PCR for transformation. The protoplasts of *A. solani* were prepared and transformed with the PCR product of the fused fragment using PEG4000. Transformants were grown on PDA plates supplemented with hygromycin. The Δ*AsCEP19* and Δ*AsCEP20* mutants were identified by PCR assays using the primers listed in Table S1; Fig. S1 after three generations of selective cultivation.

To generate gene complementation strains, the fragment containing 5′ and 3′ flanking regions of the *AsCEP19* gene, its native promoter, and the full-length sequence of *AsCEP19* was inserted into plasmid pKN, yielding the complementation vector pKN-*AsCEP19*-C. The fragment containing 5′ and 3′ flanking regions of the *AsCEP20* gene, the native promoter, and the full-length sequence of *AsCEP20* was inserted into plasmid pKN, yielding the complementation vector pKN-*AsCEP20*-C. The fragment including 5′ and 3′ flanking regions of the *AsCEP20* gene, the native promoter, and the full-length sequence of *AsCEP20*, the 1,454 bp fragment including 3′ flanking region of the *AsCEP19* gene, and the full-length sequence of *AsCEP19* were inserted into plasmid pKN, yielding the complementation vector pKN-AsCEP19 + *AsCEP20*-C. These vectors were then transformed into the Δ*AsCEP19*, Δ*AsCEP20*, and Δ*AsCEP19 + AsCEP20* mutants. Putative transformants were selected for neomycin resistance and identified by PCR using the specific primers (Table S1; Fig. S1).

### Fungal isolates, plants, and culture conditions

The *A. solani* strain HWC168 was used as the WT for all experiments in this study. To determine the expression patterns of *AsCEP19* and *AsCEP20*, WT, Δ*AsCEP19*, Δ*AsCEP19*-C, Δ*AsCEP20*, Δ*AsCEP20*-C, Δ*AsCEP19 + AsCEP20*, and Δ*AsCEP19 + AsCEP20*-C strains were grown on PDA plates at 25°C in the dark, and mycelia were harvested after 8 days.

To compare differences in pathogenicity, conidial suspensions of the WT, Δ*AsCEP19*, Δ*AsCEP19*-C, Δ*AsCEP20*, Δ*AsCEP20*-C, Δ*AsCEP19 + AsCEP20*, and Δ*AsCEP19 + AsCEP20*-C were prepared separately. Strains were grown on TA plates in the dark at 25°C for 8 days, and aerial hyphae were scraped off with a scalpel. The plates were subsequently exposed to UV light for 10 min and then kept in the dark at 25°C/20°C (12 h/12 h) for 4 days. To obtain conidial suspensions, conidia were harvested with sterile double-distilled (dd)H_2_O and centrifuged at 1,970 *g* for 10 min, and then were diluted to 10^5^ conidia/mL. Potato plants (cv Favorita) were grown in a greenhouse at 24°C with 16/18 h light-dark cycles for 8 weeks. Tomato (cv Monkeymaker) and chili pepper (cv Chaotianjiao) plants were grown in a greenhouse at 24°C with 16/18 h light-dark cycles.

### Gene expression analyses of *AsCEP19* and *AsCEP20*, and conidiogenesis regulatory genes expression using RT-qPCR

The expression levels of *AsCEP19* and *AsCEP20* were determined at different stages of infection. Potato leaves were surface-sterilized with 70% ethanol for 30 s, rinsed three times with sterile ddH_2_O, dried on filter paper, and transferred to wet filter papers placed on 1% water agar in Petri dishes. Leaves were inoculated with 20 µL of *A. solani* conidial suspension. RNA was extracted from mycelia grown on PDA and also from detached potato leaves inoculated with conidia at 48 and 72 hpi using EasyPure Plant RNA Kit (TransGen, Beijing, China), and then treated with DNase I (TransGen). First-strand cDNA was synthesized from mRNA using TransScript First-Strand cDNA Synthesis SuperMix (TransGen) according to the manufacturer’s suggestions. The gene encoding β-actin (*ACTB*) was used as an internal control. The cDNA was used as template in qPCR and was performed on a C1000 thermal cycler equipped with a CFX96 real-time PCR detection system (Bio-Rad, CA, USA). PCR was performed with MagicSYBR Mixture (CWBIO, China) and specific primers (Table S3). Relative gene expression in the samples was calculated by the ddCt method (40).

The expression of conidiogenesis regulatory gene expressions *flbA*, *abaA*, *wetA*, *abr2,* and *chsA* were measured in WT, Δ*AsCEP19*, Δ*AsCEP19*-C, Δ*AsCEP20*, Δ*AsCEP20*-C, Δ*AsCEP19 + AsCEP20*, and Δ*AsCEP19 + AsCEP20*-C strains grown on TA. RNA was extracted from mycelia grown on TA at 15 days, 21 days, and 25 days using EasyPure Plant RNA Kit (TransGen, Beijing, China), and then treated with DNase I (TransGen). First-strand cDNA was synthesized from mRNA using TransScript First-Strand cDNA Synthesis SuperMix (TransGen) according to the manufacturer’s suggestions. The *ACTB* was used as an internal control. qPCR was performed on a C1000 thermal cycler equipped with a CFX96 real-time PCR detection system (Bio-Rad, CA, USA). PCR was performed with MagicSYBR Mixture (CWBIO, China) and specific primers (Table S3). Relative gene expression for the samples was calculated by the ddCt method (40).

### Biological phenotypic analysis

The *A. solani* wild type, Δ*AsCEP19*, Δ*AsCEP19*-C, Δ*AsCEP20*, Δ*AsCEP20*-C, Δ*AsCEP19 + AsCEP20*, and Δ*AsCEP19 + AsCEP20*-C strains were measured after growth on PDA at 25°C for 6 days. The diameter of mycelia was measured in four directions every day. To measure conidial production, strains were grown on TA plates at 21 days and 25 days, and then 3 mL ddH_2_O was added to each plate to resuspend the conidia. The conidial suspensions were diluted 1: 5, and then 1 µL of each conidial suspension was placed under a 100× microscope to count the number of conidia. This was repeated nine times per plate. The sum of conidia observed in one field of view is presented on the ordinate (Fig. 3d). Data were analyzed using one-way analysis of variance, with Bonferroni *P*-value adjustment. To observe conidia morphology, conidial suspensions of the WT, Δ*AsCEP19*, Δ*AsCEP19*-C, Δ*AsCEP20*, Δ*AsCEP20*-C, Δ*AsCEP19 + AsCEP20*, and Δ*AsCEP19 + AsCEP20*-C were prepared separately. Strains were grown on TA plates in the dark at 25°C for 8 days, and aerial hyphae were scraped off with a scalpel. The plates were subsequently exposed to UV light for 10 min and then kept in the dark at 25°C/20°C (12 h/12 h) for 4 days. To obtain conidial suspensions, conidia were harvested with sterile ddH_2_O and centrifuged at 1,970 *g* for 10 min, and then were diluted to 10^5^ conidia/mL. Conidia were observed under the OLYMPUS CX31 microscope (KUY NICE, China) with a 400× objective lens. Each strain was observed in 20 fields, obtaining the total number of conidia and the number of conidia deformities for each field. Data were analyzed using the Wilcoxon test, with Bonferroni *P*-value adjustment.

### Pathogenicity assay

To compare the virulence between mutant Δ*AsCEP19*, Δ*AsCEP20*, Δ*AsCEP19 + AsCEP20* strains, complementation strains, and WT in the host potato and solanaceous host tomato, potato and tomato leaves (*n* = 30) were surface-sterilized with 70% ethanol for 30 s, rinsed three times with sterile ddH_2_O, dried on filter paper, and transferred to wet filter papers placed on 1% water agar in Petri dishes. Leaves were inoculated with 20 µL of *A. solani* conidial suspension (10^5^ conidia/mL) prepared from the different strains. The sizes of necrosis lesions were measured at 5 days after inoculation, and paired *t*-test was performed in R v.4.1.3 to determine whether there was a significant difference in virulence between the different strains. Similar results were obtained in two independent experiments.

### Construction of AsCEP19-GFP and AsCEP20-GFP mutants

The GFP cassette (Fig. S2) was constructed by fusing the *AsCEP19* or *AsCEP20* cDNA sequence with their native promoters, linker sequence (ggcgcgccgccggtggcgact), and GFP. The 5′ flanking region and the GFP cassette were ligated in pKN, and transformed into the Δ*AsCEP19*, Δ*AsCEP19 + AsCEP20*, and Δ*AsCEP20* strains to generate complementation strains. To generate the GFP strains, the 5′ flanking region of the *AsCEP19* gene (*AsCEP19LR*) and 5′ flanking region of the *AsCEP20* gene (*AsCEP20LR*) were amplified from *A. solani* genomic DNA with primer pairs 19cDNA-LR-F/R and 20cDNA-LR-F/R, respectively (Table S4). *AsCEP19* and *AsCEP20* cDNA sequences were amplified from the vector pCAMBIA1301-GFP with primers 19cDNA-linker-F/R and 20cDNA-linker-F/R, respectively (Table S4). The *GFP* gene was amplified from the vector pCAMBIA1301-GFP with primers 19cDNA-GFP-F/R and 20cDNA-GFP-F/R, respectively (Table S4). All PCR reactions were performed using Super Pfx DNA polymerase (CWBIO, China). Three PCR amplicons were fused in the order “*AsCEP19LR-AsCEP19-GFP*” and “*AsCEP20LR-AsCEP20-GFP*” and then cloned into a pKN vector using the pEASY-Uni Seamless Cloning and Assembly Kit (TransGen, Beijing, China), yielding the recombinant vectors pKN-*AsCEP19LR-AsCEP19-GFP* and pKN-*AsCEP20LR-AsCEP20-GFP*. The protoplasts of Δ*AsCEP19* and Δ*AsCEP19* + AsCEP20 mutants were prepared and transformed with recombinant vectors pKN-*AsCEP19LR-AsCEP19-GFP* (Δ*AsCEP19*::AsCEP19-GFP and Δ*AsCEP19 + AsCEP20*::AsCEP19-GFP), respectively. The protoplasts of Δ*AsCEP20* and Δ*AsCEP19* + AsCEP20 mutant were prepared and transformed with recombinant vectors pKN-*AsCEP20LR-AsCEP20-GFP* (Δ*AsCEP20*::AsCEP20-GFP and Δ*AsCEP19 + AsCEP20*::AsCEP20-GFP), respectively. The transformants were cultured on neomycin-resistant PDA for three generations, and Δ*AsCEP19*::AsCEP19-GFP, Δ*AsCEP19 +AsCEP20*::AsCEP19-GFP, Δ*AsCEP20*::AsCEP20-GFP, and Δ*AsCEP19 + AsCEP20*::AsCEP20-GFP mutants were identified by PCR using specific primers (Table S4; Fig. S3).

### Subcellular localization analysis

Determining the localization of effectors in host plant cells can reveal clues to their virulence function. Chili pepper leaves were surface-sterilized with 70% ethanol for 30 s, rinsed three times with sterile ddH_2_O, dried on filter paper, and transferred to wet filter papers placed on 1% water agar in Petri dishes. Leaves were inoculated with 20 µL of *A. solani* conidial suspensions (10^5^ conidia/mL) prepared from the Δ*AsCEP19*::AsCEP19-GFP, Δ*AsCEP19 + AsCEP20*::AsCEP19-GFP, Δ*AsCEP20*::AsCEP20-GFP, and Δ*AsCEP19 + AsCEP20*::AsCEP20-GFP strains and WT. The inoculated leaves were visualized using the FluoView FV10i fluorescence microscope at 0 days and 3 days post inoculation.

### WGCNA analysis

To explore the host immunity and metabolic pathways involved in AsCEP20, nine potato varieties with different resistance levels to *A. solani* were selected and analyzed by WGCNA. These nine potato varieties include high-resistance varieties V7, Beifang 016, and Beifang 018, and high-susceptible varieties Minshu 1, Zihuabai, Beifang001, Zhongshu 5, Atlantic, and Favorita. The middle canopy of the nine potato varieties were separately sprayed with 1 mL of spore suspension from WT and Δ*AsCEP20* strains. Potato leaves were cut off at 48 hpi and sequencing was performed using Illumina NovaSeq 6000. Raw RNA-Seq data were deposited to the Sequence Read Archive of the National Center for Biotechnology Information with BioProject ID PRJNA978953 (Table S5). Adaptor and quality trimming of raw data sets were performed using fastp v0.20.1 (41). Reads survived from adaptor and quality trimming was 98.30%. The trimmed reads of each sample were mapped to the corresponding *Solanum tuberosum* genomes (Table S5) using HISAT2 v.2.1.0 (42). The average overall alignment rate was 76.66%. Mapping results were converted to BAM format and then sorted using Picard v.2.26.11. Reads counts for each gene were calculated using the htseq-count tool in HTSeq v.0.12.4 (43). The most differentially expressed 6,000 genes (20%) were selected to construct the WGCNA network. The soft power was selected as 8, minModuleSize was 25, and MEDissThres was less than 85% to obtain each module. Association analysis of phenotypes allows the selection of modules that are significantly related to genotype. GO and KEGG pathway enrichment analyses were carried out on the genes in this module, 30 genes with high internal correlation were selected to construct a network, and the hub genes were identified. The network was visualized by VisANT v.5.0.

## SUPPLEMENTAL MATERIAL

Supplemental figures spectrum.04214-23-s0001.xlsx Fig. S1-S4.

Supplemental tables spectrum.04214-23-s0002.docx Tables S1-S5.

ASM does not own the copyrights to Supplemental Material that may be linked to, or accessed through, an article. The authors have granted ASM a non-exclusive, world-wide license to publish the Supplemental Material files. Please contact the corresponding author directly for reuse.
