# Supplementary material for: Alternaria solani effectors AsCEP19 and AsCEP20 reveal novel functions in pathogenicity and conidiogenesis
Source: Microbiol Spectr. 2024 Jun 24;12(8):e04214-23. doi: 10.1128/spectrum.04214-23 (PMC11302675; doi:10.1128/spectrum.04214-23)
Supplement: Supplemental tables — Tables S1-S5. [file spectrum.04214-23-s0002.docx]

**SUPPLEMENTAL FILE 2**

**
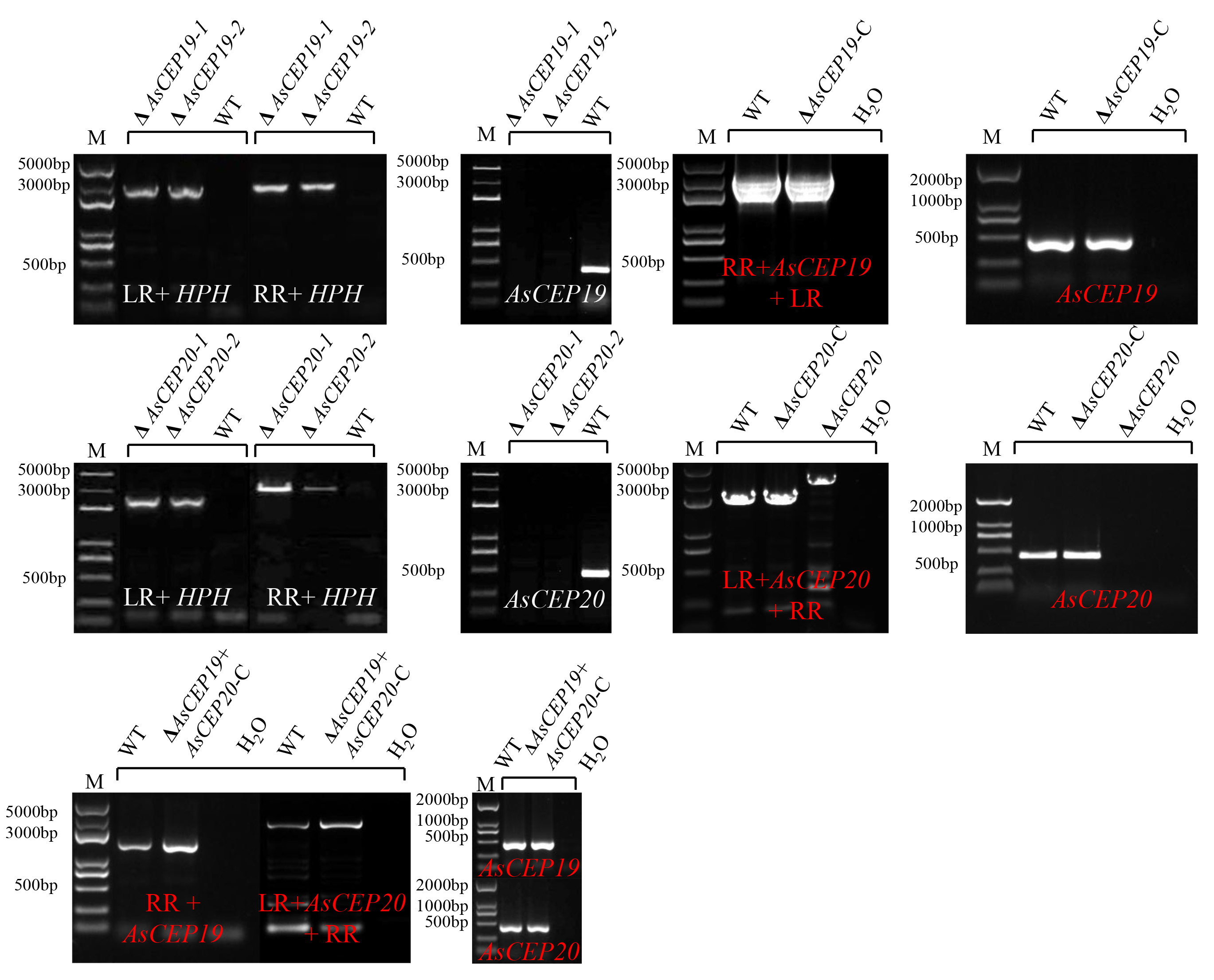
**

**Figure S1.** PCR analysis of *A. solani* mutants and complementation strains. The white words represent amplification results of these fragments in the gene knockout mutants, and the red words represent amplification results of these fragments in the complementation strains.


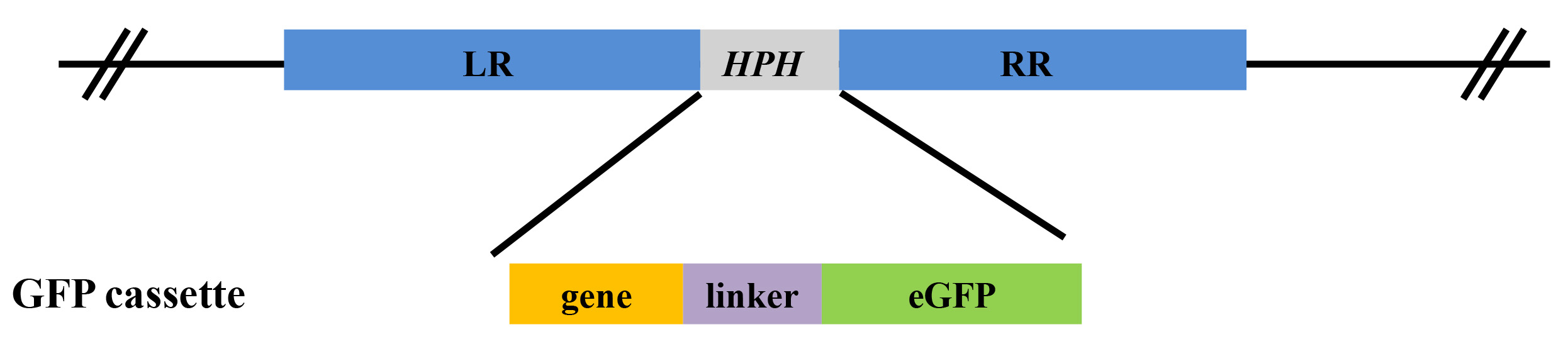


**Figure S2.** Generation of *AsCEP19* and *AsCEP20* complementation strain by protoplast transformation of the Δ*AsCEP19*, Δ*AsCEP19*+*AsCEP20* and Δ*AsCEP20* strains with the native *AsCEP19* and *AsCEP20* fragment and GFP cassette.


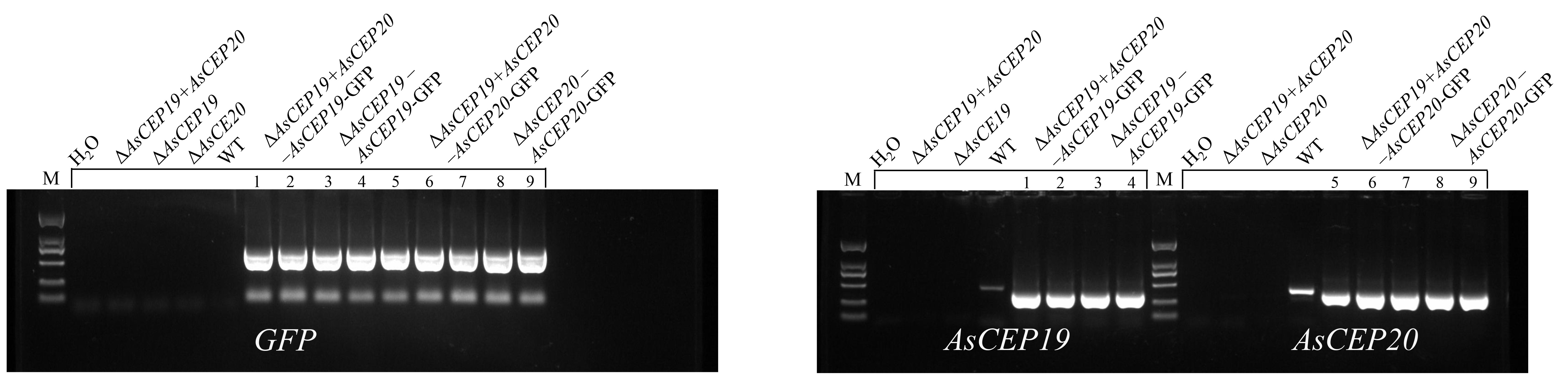


**Figure S3.** PCR analysis of the Δ*AsCEP19*::AsCEP19-GFP, Δ*AsCEP19*+*AsCEP20*::AsCEP19-GFP, Δ*AsCEP20*::AsCEP20-GFP and Δ*AsCEP19*+*AsCEP20*::AsCEP20-GFP strains. The first to third lanes represent the amplification results of *GFP* gene sequence and *AsCEP19* gene cDNA sequence in the Δ*AsCEP19+AsCEP20*. The fourth lanes represent the amplification results of *GFP* gene sequence and *AsCEP19* gene cDNA sequence in Δ*AsCEP19*. The fifth to eighth lanes represent the amplification results of *GFP* gene sequence and *AsCEP20* gene cDNA sequence in the Δ*AsCEP19+AsCEP20*. The ninth lanes represent the amplification results of *GFP* gene sequence and *AsCEP20* gene cDNA sequence in Δ*AsCEP20*.


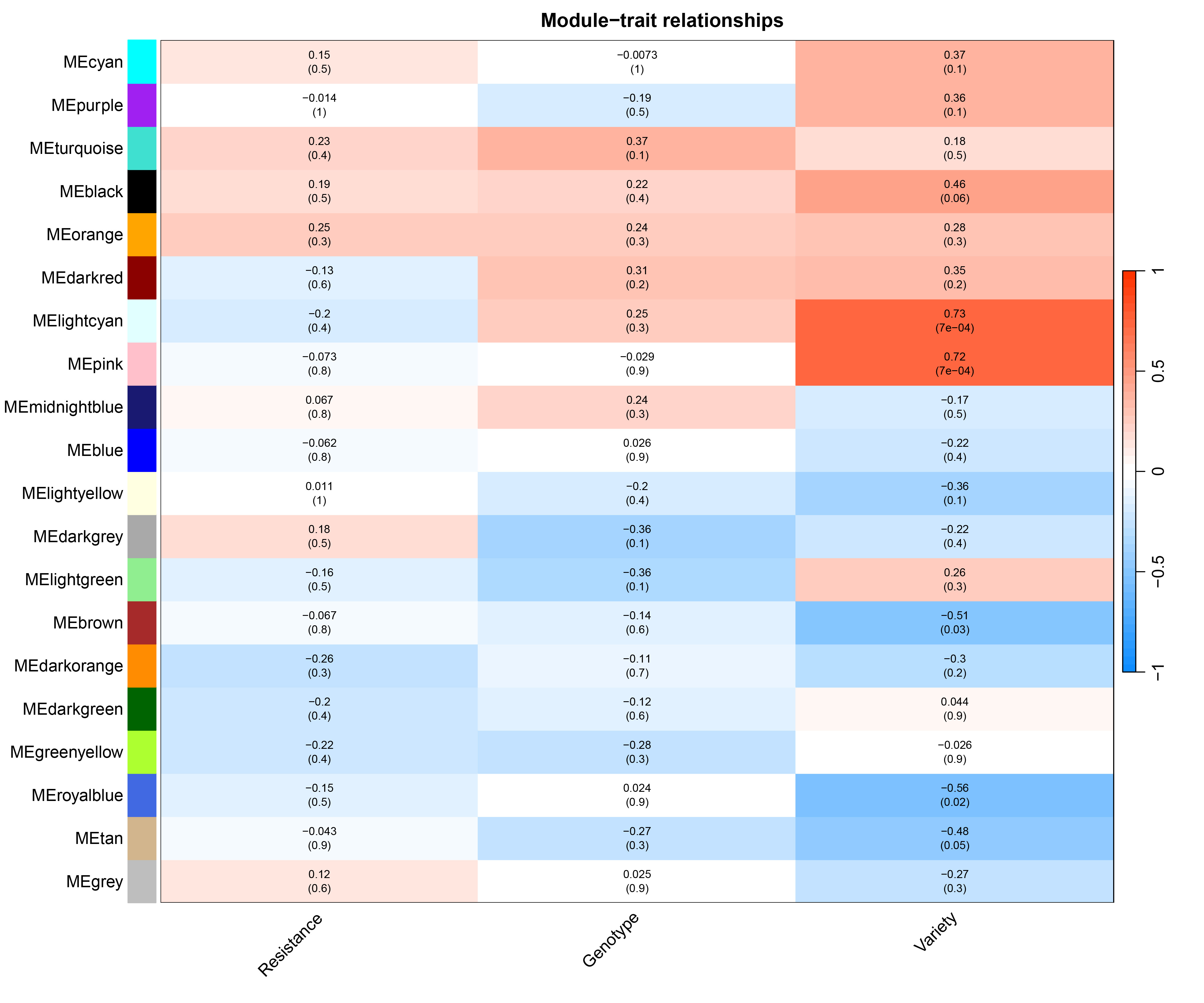


**Figure S4.** The relationship diagram between different gene modules and potato resistance, strain genotype and potato variety.
